# The neurophysiological lesson from the Italian CIDP database

**DOI:** 10.1007/s10072-021-05321-z

**Published:** 2021-05-21

**Authors:** Emanuele Spina, Pietro Emiliano Doneddu, Giuseppe Liberatore, Dario Cocito, Raffaella Fazio, Chiara Briani, Massimiliano Filosto, Luana Benedetti, Giovanni Antonini, Giuseppe Cosentino, Stefano Jann, Anna Mazzeo, Andrea Cortese, Girolama Alessandra Marfia, Angelo Maurizio Clerici, Gabriele Siciliano, Marinella Carpo, Marco Luigetti, Giuseppe Lauria, Tiziana Rosso, Guido Cavaletti, Erdita Peci, Stefano Tronci, Marta Ruiz, Stefano Cotti Piccinelli, Angelo Schenone, Luca Leonardi, Luca Gentile, Laura Piccolo, Giorgia Mataluni, Lucio Santoro, Eduardo Nobile-Orazio, Fiore Manganelli, on the behalf of the Italian CIDP Database Study Group

**Affiliations:** 1 Department of Neuroscience, Reproductive Sciences and Odontostomatology, University of Naples ‘Federico II’, Via Pansini, 5, 81025 Naples, Italy; 2 Neuromuscular and Neuroimmunology Service, Humanitas Clinical and Research Institute, Rozzano, Milan, Italy; 3 Presidio Sanitario Major, Istituti Clinici Scientifici Maugeri, Turin, Italy; 4 Division of Neuroscience, Department of Neurology, Institute of Experimental Neurology (INSPE), San Raffaele Scientific Institute, Milan, Italy; 5 Neurology Unit, Department of Neuroscience, University of Padova, Padova, Italy; 6 Center for Neuromuscular Diseases and Neuropathies, Unit of Neurology, ASST ‘Spedali Civili’, University of Brescia, Brescia, Italy; 7 Department of Neuroscience, Rehabilitation, Ophthalmology, Genetics, Maternal and Child Health, University of Genoa and IRCCS San Martino, Genoa, Italy; 8 IRCCS AOU San Martino-IST, Genoa, Italy; 9 Unit of Neuromuscular Diseases, Department of Neurology Mental Health and Sensory Organs (NESMOS), Faculty of Medicine and Psychology, ‘Sapienza’ University of Rome, Sant’Andrea Hospital, Rome, Italy; 10 Department of Brain and Behavioral Sciences, University of Pavia, Pavia, Italy; 11 IRCCS Foundation C. Mondino National Neurological Institute, Pavia, Italy; 12 Department of Neuroscience, Niguarda Ca’ Granda Hospital, Milan, Italy; 13 Department of Clinical and Experimental Medicine, Unit of Neurology, University of Messina, Messina, Italy; 14 Molecular Neurosciences, University College London, London, UK; 15 Dysimmune Neuropathies Unit, Department of Systems Medicine, Tor Vergata University of Rome, Rome, Italy; 16 Neurology Unit, Circolo & Macchi Foundation Hospital, Insubria University, DBSV, Varese, Italy; 17 Neurology Unit, Department of Clinical and Experimental Medicine, University of Pisa, Pisa, Italy; 18 Neurology Unit, ASST Bergamo Ovest-Ospedale Treviglio, Treviglio, Italy; 19 Fondazione Policlinico Universitario A. Gemelli IRCCS, UOC Neurologia, Università Cattolica del Sacro Cuore, Rome, Italy; 20 Unit of Neuroalgology, IRCCS Foundation ‘Carlo Besta’ Neurological Institute, Milan, Italy; 21 Department of Biomedical and Clinical Sciences ‘Luigi Sacco’, University of Milan, Milan, Italy; 22 ULSS2 Marca Trevigiana, UOC Neurologia-Castelfranco Veneto, Treviso, Italy; 23 School of Medicine and Surgery and Experimental Neurology Unit, University of Milano-Bicocca, Monza, Italy; 24 Department of Medical Biotechnology and Translational Medicine, Milan University, Milan, Italy

**Keywords:** CIDP, Neurophysiology, Diagnostic criteria, Nerve conduction

## Abstract

**Introduction:**

Electrophysiological diagnosis of chronic inflammatory demyelinating polyradiculoneuropathy (CIDP) may be challenging. Thus, with the aim ofproviding some practical advice in electrophysiological approach to a patient with suspected CIDP, we analyzed electrophysiological data from 499 patients enrolled inthe Italian CIDP Database.

**Methods:**

We calculated the rate of each demyelinating feature, the rate of demyelinating features per nerve, the diagnostic rate for upper andlower limb nerves, and, using a ROC curve analysis, the diagnostic accuracy of each couple of nerves and each demyelinating feature, for every CIDP subtype.Moreover, we compared the electrophysiological data of definite and probable CIDP patients with those of possible and not-fulfilling CIDP patients, and by a logisticregression analysis, we estimated the odds ratio (OR) to make an electrophysiological diagnosis of definite or probable CIDP.

**Results:**

The ulnar nerve had the highestrate of demyelinating features and, when tested bilaterally, had the highest diagnostic accuracy except for DADS in which peroneal nerves were the most informative.In possible and not-fulfilling CIDP patients, a lower number of nerves and proximal temporal dispersion (TD) measurements had been performed compared to definiteand probable CIDP patients. Importantly, OR for each tested motor nerve and each TD measurement was 1.59 and 1.33, respectively.

**Conclusion:**

Our findingsdemonstrated that the diagnosis of CIDP may be missed due to inadequate or incomplete electrophysiological examination or interpretation. At the same time, thesedata taken together could be useful to draw a thoughtful electrophysiological approach to patients suspected of CIDP.

**Supplementary Information:**

The online version contains supplementary material available at 10.1007/s10072-021-05321-z.

## Introduction

Chronic inflammatory demyelinating polyradiculoneuropathy (CIDP) is a chronic and disabling disease with a range prevalence of 0.8–8.9 cases per 100,000 and incidence of 0.2–1.6/100,000 [1]. It is the most frequent acquired immune-mediated chronic neuropathy and has a broad spectrum of possible clinical presentation including typical and atypical forms [2–4].

Diagnosis of CIDP is made by demonstrating peripheral nerve demyelination, commonly by electrophysiological testing. Hence, several sets of electrophysiological criteria for diagnosis of CIDP have been proposed, and the most widely accepted criteria are those recommended by the European Federation of Neurological Societies and Peripheral Nerve Society (EFNS/PNS). Based on the number and electrophysiological features (i.e., prolonged distal motor latency (DML), motor nerve conduction velocity (MNCV) slowing, temporal dispersion (TD), probable or definite conduction block (CB), and absent or prolonged F-wave), three diagnostic electrophysiological classes are identifiable: definite, probable, and possible CIDP [5–7].

The diagnosis may be easy when demyelinating signs are widespread, while it may be challenging when demyelination is limited to few or specific nerves or nervous tracts (e.g., proximally) [8–10].

Accordingly, in clinical practice, neurophysiological examination in search of demyelinating signs may be time-consuming and eventually not decisive, so making tricky the diagnosis and delaying the start of a proper treatment.

Thus, having the opportunity to access the Italian CIDP Database [3, 4], we have analyzed a large amount of electrophysiological data. Our aim was to obtain a wide overview of the electrophysiological characteristics of CIDP that could provide some practical advice in electrophysiological approach to a patient with suspected CIDP.

Moreover, we analyzed the electrophysiological data from patients who, although diagnosed as having CIDP based on clinical and supportive features, did not fulfill the electrophysiological criteria for demyelination.

## Patients and methods

This was a retrospective multicenter cohort study in a large sample of CIDP patients implementing a web database (CINECA, Bologna, Italy) to collect demographical, clinical, and electrophysiological data from patients diagnosed and followed by 22 centers throughout Italy with expertise on CIDP.

The ethical committee of each participating center approved the study and all the patients gave written informed consent.

Clinical and neurophysiological data were obtained by an experienced neurologist with a neuromuscular subspecialty. Verification of the diagnostic data for all of the enrolled patients was centralized in the database coordinator center (Humanitas). Data monitoring included diagnosis revision, suspect double entries, missing data, and plausibility checks. Patients with an alternative diagnosis for the neuropathy, increased titers of anti-myelin-associated glycoprotein IgM antibodies, or without available nerve conduction studies were excluded [11].

For the current study, all electrophysiological data (blinded compared to clinical and laboratory data) were further evaluated independently and after discussed all together by three expert neurophysiologists (ES, LS, FM) in the center of Naples. Based on EFNS/PNS criteria for demyelination, patients were classified into four diagnostic categories: “definite,” “probable,” “possible,” and “not-fulfilling” electrophysiological diagnosis of CIDP. Afterward, the electrophysiological results were discussed with the coordinating center (ENO, PD) and combined with clinical and laboratory data. The neurophysiological study was contemporaneous with clinical classification of CIDP.

We calculated in CIDP patients (definite, probable, and possible) the rate of each demyelinating feature, the rate of demyelinating features per nerve (median, ulnar, peroneal, and tibial), and the diagnostic rate as established by EFNS/PNS for upper and lower limb nerves. To exclude median neuropathy at the wrist (carpal tunnel syndrome), distal motor latency in the median nerve was not considered in our analysis when associated with slowing of sensory nerve conduction velocities or absence of sensory action potential.

We ran a receiver operating characteristic (ROC) curve analysis to measure the diagnostic accuracy for each possible couple of nerves and for each demyelinating feature for CIDP diagnosis (definite, probable, or possible). For ROC curve analysis, we used as diagnostic gold standard the diagnosis of CIDP based on the combination of clinical features, neurophysiology data, and supportive criteria.

We also ran ROC curve analysis separately for typical CIDP and for each atypical CIDP subtype (i.e., multifocal acquired demyelinating sensory and motor (MADSAM), distal acquired demyelinating symmetric (DADS), pure sensory ,and pure motor). Values for the area under the curve (AUC) obtained from ROC curve analysis above 0.8 were considered excellent [12].

Moreover, we compared the electrophysiological approach (i.e., number of tested motor nerves and number of each electrophysiological measure per patient) among the patients with definite and probable CIDP and those with possible CIDP and not fulfilling electrophysiological CIDP criteria. We decided to analyze together possible and not-fulfilling CIDP patients as “possible” category is a very poorly specific category [5].

Thus, through a logistic regression analysis, we estimated the odds ratio (OR) of differences in electrophysiological approach applied to patients with definite and probable CIDP with respect to that applied to possible and not-fulfilling groups and then we used a multivariable logistic regression analysis for estimating the independent weight of differences emerged from the univariate logistic regression analysis.

Lastly, we evaluated the percentage of diagnosed patients (definite and probable) according to the number of tested nerves (2, 3, 4, 5, 6, 7, and 8 nerves) and the number of proximal temporal dispersion measurements. After finding that the percentage of CIDP diagnosis progressively increased up to six tested nerves while after (more than six nerves) the diagnostic rate remained unchanged, the value of 6 (nerves) was set as a cut-off and as a reference value for a regression analysis.

Each regression analysis was controlled for age and sex, and odds ratios and 95% confidence intervals were reported.

### Ethics approval

The study was approved by the ethical committee of each participating center.

### Consent to participate

Written informed consent was obtained from all participants at enrollment.

### Data availability Policy

Any data not published within this article is fully available previous anonymization by request from a qualified investigator.

## Results

We analyzed data from 499 (out of 545) patients included consecutively into the Italian CIDP Database from January 2015 to January 2019. Forty-four patients were excluded from the analysis for the presence of a different cause for neuropathy or unavailable neurophysiological data [11]. Two patients with chronic immune sensory polyradiculopathy (CISP) were excluded from the analysis [13].

First, we divided our dataset according to electrophysiological data: 352/499 patients (70.6%) were classified as definite CIDP, 10/499 (2%) as probable CIDP, 57/499 (11.4%) as possible CIDP, and 80/499 (16%) as CIDP not fulfilling the EFNS/PNS electrophysiological criteria [6]. Then, we evaluated the subtype: typical patients were 397/499 (79.7%); MADSAM 18/499 (3.4%), DADS 42/499 (8.4%), 20/499 pure motor (4%), 17/499 pure sensory (3.4%), and 5/499 focal (1%) (Supplementary Table 1).

For patients that did not meet EFNS/PNS electrophysiological criteria for demyelination, the diagnosis of CIDP was discussed among the neurophysiologist panel, the database coordinator center, and the physician who had enrolled the patient. Therefore, these patients were considered affected by CIDP because, though they did not have nerve conduction abnormalities (Supplementary Table 2), satisfying EFNS/PNS criteria, nevertheless, they showed clinical, laboratory, and neurophysiological features (i.e., clinical relapse, response to therapies, elevated cerebrospinal fluid proteins, magnetic resonance imaging with gadolinium enhancement, nerve biopsy, somatosensory-evoked potentials) supportive of CIDP.

In definite, probable, and possible CIDP patients as a whole, DML was prolonged in 160/419 (38.2%), MNCV was reduced in 285/419 (68%), proximal TD was present in 183/419 (43.7%), probable and definite CBs were observed in 46/419 (11%) and 134/419 (32%), respectively, and F-wave was absent or prolonged in 145/419 (34.6%) patients. Prolonged distal compound muscle action potential (CMAP) duration was excluded from this analysis because of heterogeneity of data.

The highest rate of demyelinating features was observed in the ulnar nerve (right= 67.9%; left= 69.3%). The frequency for each demyelinating feature per nerve (median, ulnar, peroneal, and tibial) is reported in Table 1.

**Table 1:** Rate of demyelinating features per nerve

Nerve	Diagnostic class	Studied nerves/patients	Prolonged DML	NCV slowing	Temporal Dispersion	CBs (probable; definite)	F-wave alteration	Nerves with at least one demyelinating feature
Right median nerve	Definite	253/352 (71.8%)	27/253 (10.7%), 51 excluded	120/253 (47.4%)	41/253 (16.2%)	26/253 (10.2%); 28/253 (11.1%)	44/253 (17.4%)	176/253 (69.6%)
	Probable	8/10 (80%)	0/8	0/8	0/8	3/8 (37.5%); 0/8	0/8	3/8 (37.5%)
	Possible	35/57 (61.4%)	2/35 (5.7%), 4 excluded	5/35 (14.3%)	0/35	2/35 (5.7%); 2/35 (5.7%)	2/35 (5.7%)	9/35 (25.7%)
	Total	296/419 (70.6%)	29/296 (9.8%), 55 excluded	125/296 (42.2%)	41/296 (13.8%)	31/296 (10.4%); 30/296 (10.1%)	46/296 (15.5%)	188/296 (63.5%)
Left median nerve	Definite	216/352 (61.4%)	23/216 (10.6%), 44 excluded	104/216 (48.1%)	42/216 (19.4%)	16/216 (7.4%); 26/216 (12%)	33/216 (15.3%)	152/216 (70.4%)
	Probable	7/10 (70%)	0/7	0/7	0/7	3/7 (42.8%); 0/7	0/7	3/7 (42.8%)
	Possible	19/57 (33.3%)	1/19 (5.3%), 1 excluded	0/19	0/19	0/19 0/19	0/19	1/19 (5.3%)
	Total	242/419 (57.5%)	24/242 (9.9%), 45 excluded	104/242 4(43%)	42/242 (17.4%)	19/242 (7.8%); 26/242 (10.7%)	33/242 (13.6%)	156/242 (64.5%)
Right ulnar nerve	Definite	271/352 (76.9%)	70/271 (25.8%)	124/271 (45.7%)	51/271 (18.9%)	29/271 (10.7%); 17/271 (6.3%)	57/271 (21%)	202/271 (74.5%)
	Probable	8/10 (80%)	0/8	0/8	0/8	1/8 (12.5%); 0/8	0/8	1/8 (12.5%)
	Possible	36/57 (63.2%)	2/36 (5.6%)	6/36 (16.7%)	2/36 (5.6%)	1/36 (2.7%); 1/36 (2.7%)	2/36 (5.6%)	11/36 (30.6%)
	Total	315/419 (75.2%)	72/315 (22.8%)	130/315 (41.3%)	53/315 (16.9%)	31/315 (9.8%); 18/315 (5.7%)	59/315 (18.7%)	214/315 (67.9%)
Left ulnar nerve	Definite	244/352 (69.3%)	69/244 (28.3%)	104/244 (42.6%)	51/244 (20.9%)	11/244 (4.5%) ; 15/244 (6.1%)	46/244 (18.9%)	182/244 (74.6%)
	Probable	8/10 (80%)	0/8	1/8 (12.5%)	1/8 (12.5%)	1/8 (12.5%); 0/8	0/8	3/8 (37.5%)
	Possible	25/57 (43.9%)	1/25 (4%)	3/25 (12%)	1/25 (4%)	0/25; 0/25	3/25 (12%)	7/25 (28%)
	Total	277/419 (66.1%)	70/277 (25.2%)	108/277 (38.9%)	53/277 (19.1%)	12/277 (4.3%); 15/277 (5.4%)	49/277 (17.6%)	192/277 (69.3%)
Right peroneal nerve	Definite	283/352 (80.4%)	44/283 (15.5%)	81/283 (28.6%)	66/283 (23.3%)	27/283 (9.5%); 24/283 (8.5%)	17/283 (6%)	170/283 (60%)
	Probable	7/10 (70%)	0/7	0/7	1/7 (14.3%)	0/7; 0/7	1/7 (14.3%)	2/7 (28.6%)
	Possible	43/57 (75.4%)	1/43 (2.3%)	3/43 (6.9%)	4/43 (9.3%)	0/43; 2/43 (4.7%)	0/43	10/43 (23.2%)
	Total	333/419 (79.5%)	45/333 (13.5%)	84/333 (25.2%)	71/333 (21.3%)	27/333 (8.1%); 26/333 (7.8%)	18/333 (5.4%)	182/333 (54.7%)
Left peroneal nerve	Definite	281/352 (79.8%)	40/281 (14.2%)	89/281 (31.7%)	58/281 (20.6%)	14/281 (4.9%); 29/281 (10.3%)	19/281 (6.7%)	174/281 (61.9%)
	Probable	9/10 (90%)	0/9	0/9	0/9	3/9 (33.3%); 0/9	1/9 (11.1%)	4/9 (44.4%)
	Possible	39/57 (68.4%)	0/39	6/39 (15.4%)	1/39 (2.6%)	0/39; 0/39	0/39	9/39 (23.1%)
	Total	329/419 (78.5%)	40/329 (12.1%)	95/329 (28.8%)	59/329 (17.9%)	17/329 (5.1%); 29/329 (8.8%)	20/329 (6.1%)	187/329 (56.9%)
Right tibial nerve	Definite	218/352 (62%)	52/218 (23.8%)	70/218 (32.1%)	61/218 (28%)	32/218 (14.7%); 0/218	32/218 (14.7%)	158/218 (72.5%)
	Probable	4/10 (40%)	0/4	0/4	2/4 (50%)	1/4 (25%)	0/4	2/4 (50%)
	Possible	27/57 (47.4%)	1/27 (3.7%)	0/27	2/27 (7.4%)	0/27	2/27 (7.4%)	5/27 (18.5%)
	Total	249/419 (59.4%)	53/249 (21.3%)	70/249 (28.1%)	65/249 (26.1%)	33/249 (13.2%)	34/249 (13.7%)	165/249 (66.2%)
Left tibial nerve	Definite	232/352 (65.9%)	54/232 (23.2%)	77/232 (33.2%)	43/232 (18.5%)	NA; 41/232 (17.7%)	37/232 (15.9%)	156/232 (67.2%)
	Probable	6/10 (60%)	0/6	0/6	1/6 (16.7%)	1/6 (16.7%)	0/6	2/6 (33.3%)
	Possible	32/57 (56.1%)	0/32 (6.3%)	2/32 (6.3%)	3/32 (9.4%)	NA; 0/32	0/32	5/32 (15.6%)
	Total	270/419 (64.4%)	54/270 (20%)	79/270 (29.2%)	47/270 (17.4%)	42/270 (15.5%)	37/270 (13.7%)	163/270 (60.4%)

Overall, at least one nerve with demyelinating features was observed in 65.7% of patients considering only the upper limbs, and in 64.5% of patients considering only the lower limbs. However, the analysis per subtypes showed that the rate of demyelinating features was comparable between upper and lower limbs for typical and sensory forms while it was higher in upper than lower limbs in MADSAM (70.6 vs 58.9%) and motor (64.7% vs 35.3%) subtypes and it was higher in lower limbs in DADS phenotype (73.8% vs 40.5%) (Table 2).

**Table 2:** Rate of patients with at least one demyelinating feature in upper and lower limbs

	Overall	Typical	MADSAM	DADS	Sensory	Motor	Focal
Upper limbs	328/499 (65.7%)	275/397 (69.3%)	12/17 (70.6%)	17/42 (40.5%)	11/20 (55%)	11/17 (64.7%)	2/5 (40%)
Lower limbs	322/499 (64.5%)	259/338 (65.2%)	10/17 (58.9%)	31/42 (73.8%)	12/20 (60%)	6/17 (35.3%)	3/5 (60%)

**Fig. 1:**
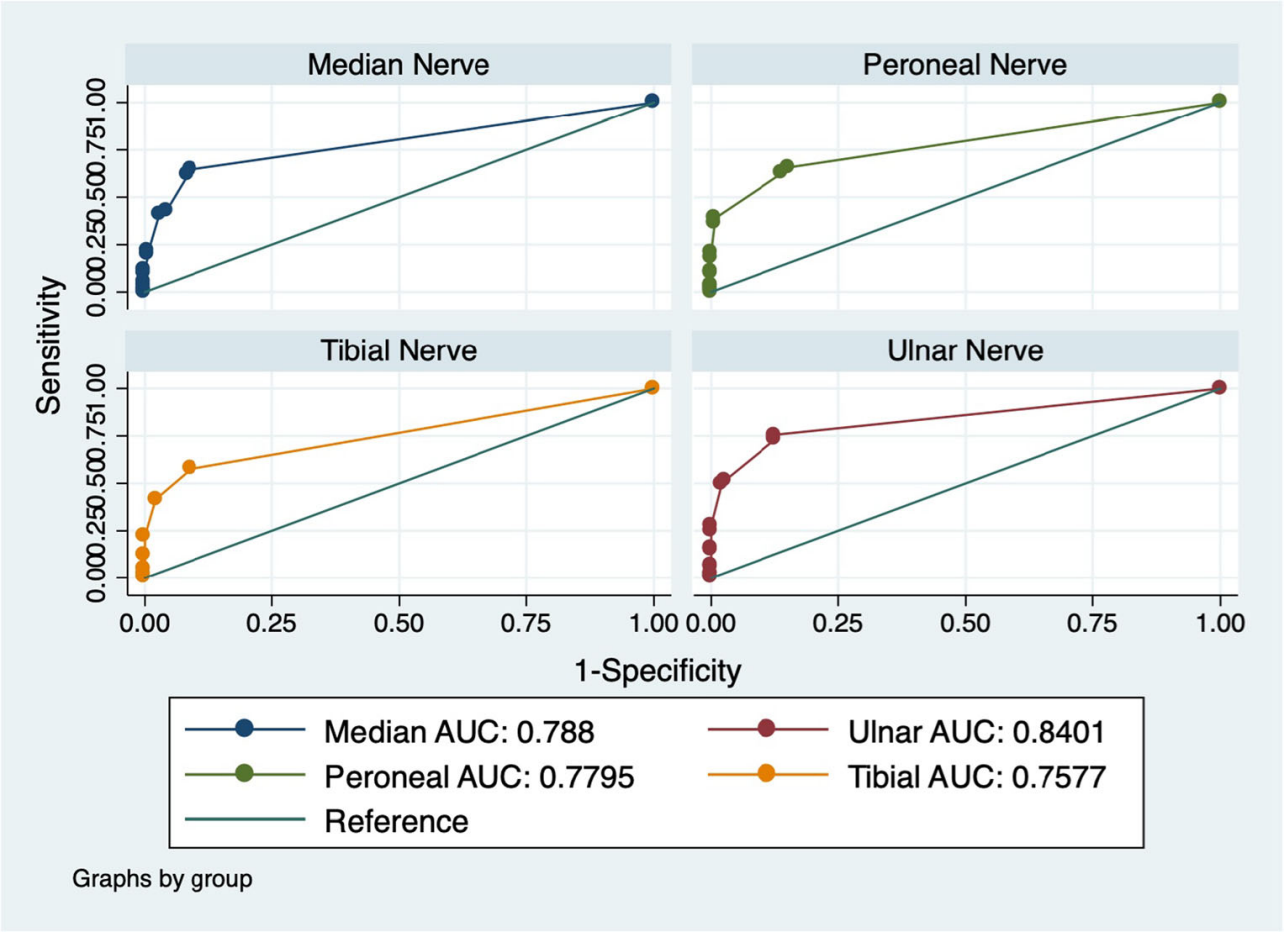
ROC curve analysis showing diagnostic accuracy for each nerve in the entire population

The ROC curve analysis for nerves showed the highest diagnostic accuracy for ulnar nerves and the lowest for tibial nerves (Table 3 and Fig. 1) in the overall population.

**Table 3:** ROC curve analysis

	Per nerve (overall)	Per couple (overall)	Per couple (typical)	Per couple (MADSAM)	Per couple (DADS)	Per couple (sensory)	Per couple (motor)
Right median nerve	0.7133	0.7880	0.8019	0.6894	0.6833	0.8	0.9
Left median nerve	0.7092						
Right ulnar nerve	0.7530	0.8401	0.8509	0.9167	0.7347	0.92	0.9157
Left ulnar nerve	0.7396						
Right peroneal nerve	0.7045	0.7795	0.7719	0.5758	0.9167	0.84	0.7
Left peroneal nerve	0.7185						
Right tibial nerve	0.7066	0.7577	0.7499	0.6667	0.8625	0.825	0.6786
Left tibial nerve	0.6929

In typical CIDP, as well in MADSAM, sensory, and motor subtypes, the ROC curve analysis showed the highest diagnostic accuracy for ulnar nerve evaluated bilaterally, followed by the combination of ulnar and peroneal nerves, independently from the side (Supplementary Table 3).

Instead, in DADS subtype, the highest diagnostic accuracy was observed for lower limb nerves and especially for peroneal nerve evaluated bilaterally, followed by the combination of peroneal and tibial (independently from the side). An excellent AUC was reported for the combination of ulnar and peroneal nerves, taken together (AUC: 0.95). Detailed data for each subtype are reported in Supplementary Table 4.

**Fig. 2:**
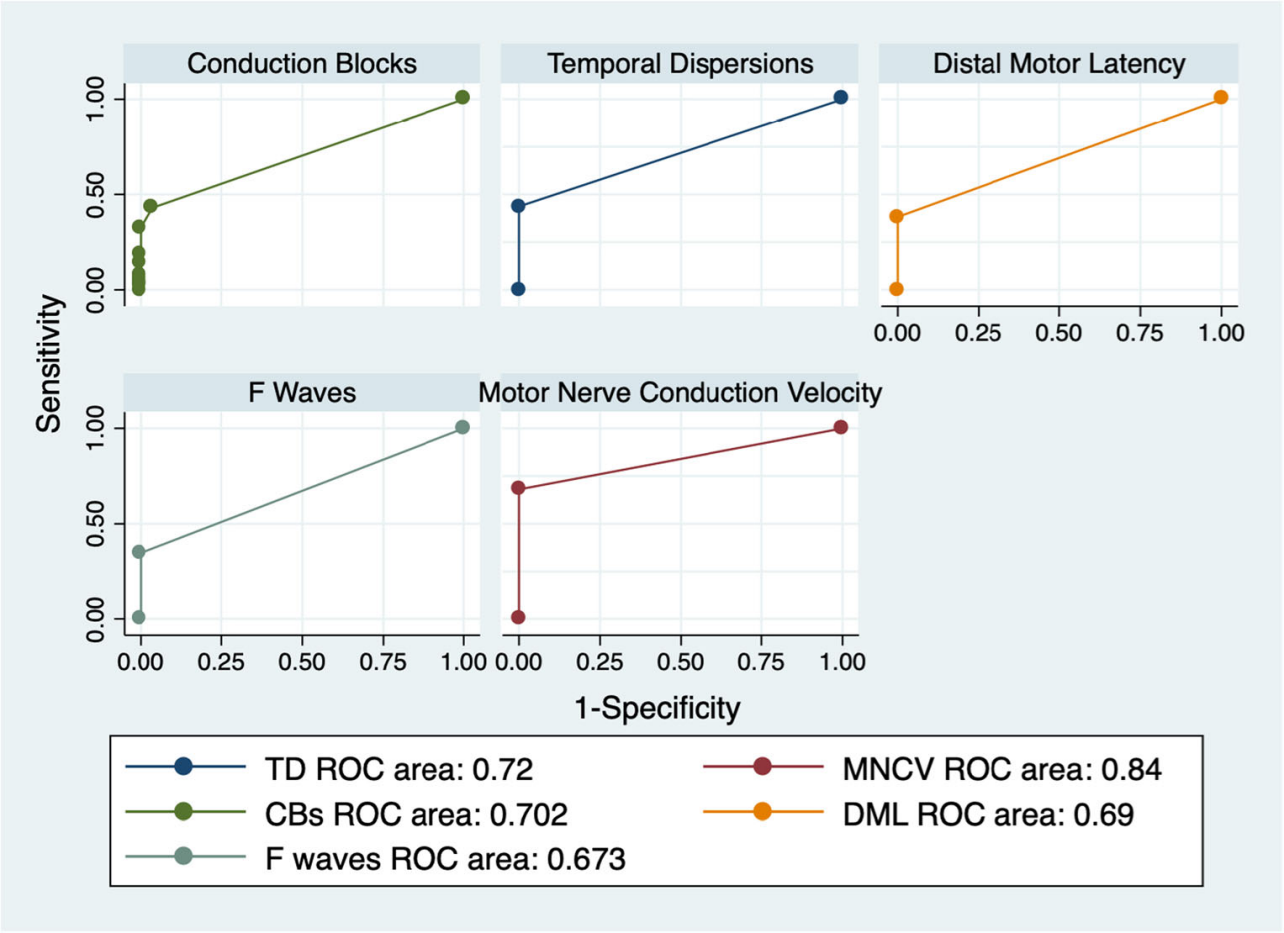
ROC curve analysis showing diagnostic accuracy for each demyelinating feature in the entire population

The ROC curve analysis for demyelinating features showed the highest diagnostic accuracy for MNCV slowing (AUC: 0.8401) (Fig. 2).

The patients with a diagnosis of definite and probable CIDP with respect to those with possible CIDP or not fulfilling electrophysiological CIDP criteria had a higher median value (6 vs 4) of tested motor nerves as a whole and also for upper (3 vs 2) and lower limbs (3 vs 2) considered separately.

In the entire cohort of patients, the median number of tested motor nerves was 6 (5.3 mean, 1.76 SD), 2 in the upper limbs (mean 2.6, 1.21 SD) and 3 in the lower limbs (mean 2.8, 1.1 SD).

In definite CIDP patients, the median number of tested motor nerves was 6 (mean 5.6, SD 1.57), 3 in the upper limbs (mean 2.8, SD 1.13) and 3 in the lower limbs (mean 2.9, SD 1.1).

In probable CIDP patients, the median number of tested motor nerves was 6 (mean 5.4, SD 1.95), 3 in the upper limbs (2.9 mean, 1.1 SD) and 3 in the lower limbs (mean 2.5, 1.2 SD).

In possible CIDP patients, the median number of tested motor nerves was 4 (4.5 mean, 2 SD), 2 in the upper limbs (2 mean, 1.2 SD) and in 2 in the lower limbs (mean 2.5, 1.2 SD).

In CIDP patients not fulfilling the diagnostic criteria, the median number of tested motor nerves was 4 (4.6 mean, 1.8 SD), 2 in the upper limbs (mean 1.9 SD 1.2) and 2 in the lower limbs (mean 2.5, SD 1.2).

**Fig. 3:**
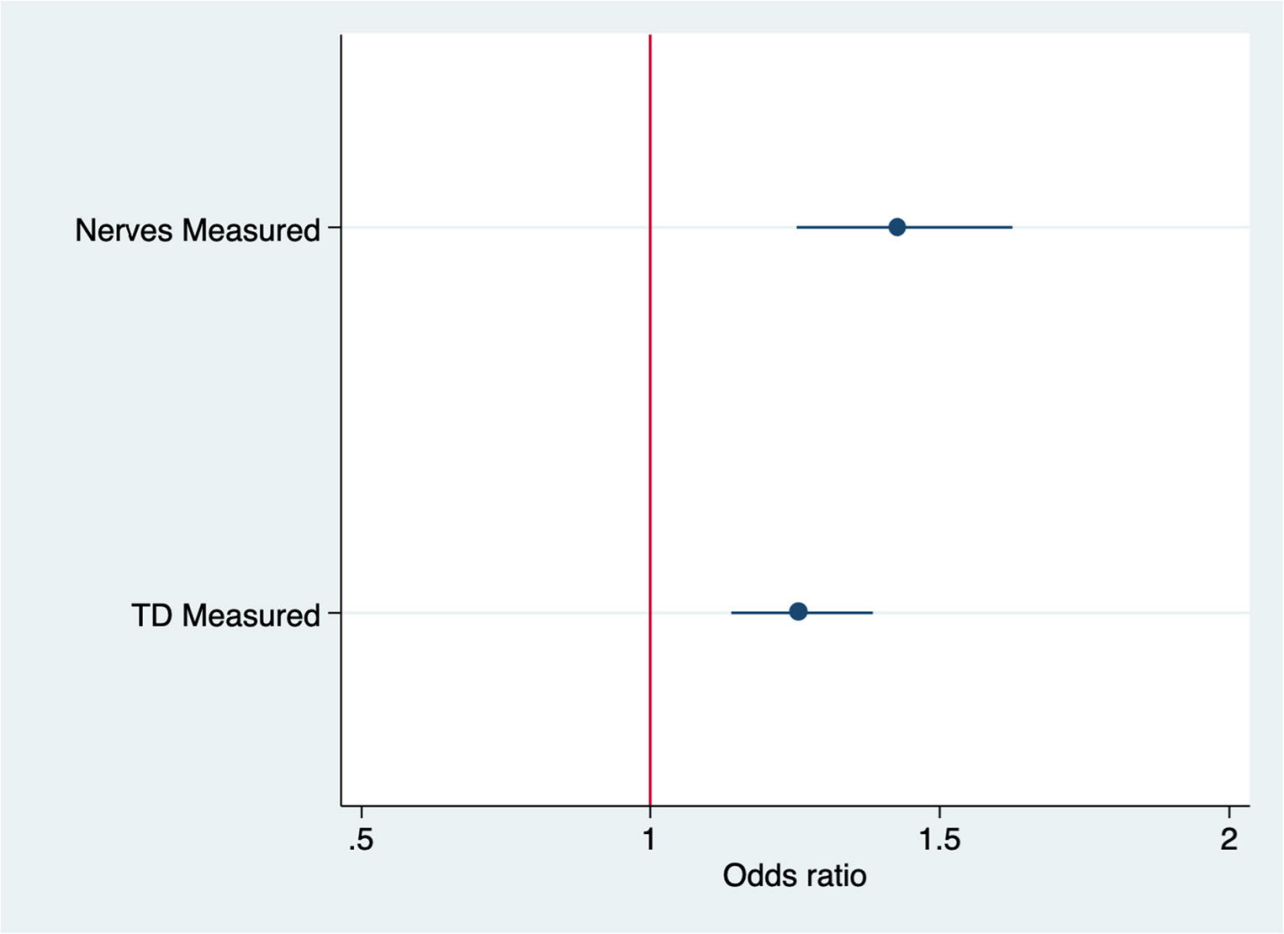
Diagnostic odds ratio for each tested nerve and proximal temporal dispersion measurement

The univariate logistic regression analysis showed an odds ratio (OR) of 1.59 (CI: 1.40–1.81; *z*: 7.2; *p*<0.00) meaning that for each nerve added to neurophysiological evaluation there was an additional 60% probability to make an electrophysiological diagnosis of definite or probable CIDP (Fig. 3). Repeating this evaluation for upper and lower limbs separately, upper limbs demonstrated higher value (OR: 1.97; CI: 1.64–2.36; *z*= 7.28; *p*<0.00) than lower limbs (OR: 1.36; CI: 1.15–1.62; *z*: 3.56; *p*<0.00).

In possible CIDP patients as well in those not fulfilling CIDP electrophysiological criteria, proximal TD was calculated less times compared to definite and probable CIDP patients (median value: 0 vs 4 times); *p*<0.00).

The logistic regression analysis demonstrated an OR of 1.33 (CI: 1.22–1.45; *z*: 6.52; *p*<0.00), meaning that for each TD measurement, there was an additional 33% probability to reach an electrophysiological diagnosis of definite or probable CIDP (Fig. 3). To exclude that this finding may simply depend on the greater number of tested motor nerves, we performed a multiple logistic regression analysis using, as independent variables, both the number of tested nerves and TD measurements, and as dependent variable the diagnostic class (CIDP (definite, probable) group vs possible and not-fulfilling criteria group), and we confirmed a significant relationship for both the independent variables (*p*<0.00 for both variables).

Lastly, regression analysis for the number of tested nerves and for the number of proximal temporal dispersion measurement showed that the study of more than 6 nerves did not further improve the diagnostic rate of definite or probable CIDP (Table 4).

**Table 4:** Distribution of percentage of diagnosed patients according to number of tested nerves and statistical comparison

Tested nerves	Number of patients	Definite + probable diagnosis (%)	Comparison with cut-off (6)
2	26	4/26 (15.4%)	6 vs 2; *p*<0.00
3	59	26/59 (44.1%)	6 vs 3; *p*<0.00
4	87	59/87 (67.8%)	6 vs 4; *p*<0.00
5	65	49/65 (75.4%)	6 vs 5; *p*=0.26
6	137	114/137 (83.2%)	Reference value
7	49	43/49 (87.7%)	6 vs 7; *p*=0.45
8	76	61/76 (80.2%)	6 vs 8; *p*=0.59
Total	499	356/499 (71.3%)	

## Discussion

We conducted an extensive analysis on the neurophysiological features in a very large cohort of CIDP patients from the Italian CIDP Database.

Nerve conduction slowing is typically considered the hallmark of demyelinating diseases; thus, it is not surprising that, among all the demyelinating features, MNCV was the most frequently altered (68% of patients) and showed the best diagnostic accuracy (AUC: 0.8401).

The most informative nerve was the ulnar nerve, which had the highest rate of demyelinating features (right= 67.9%; left= 69.3%), as well as ulnar nerve tested bilaterally, which showed the highest diagnostic accuracy in CIDP population as a whole (AUC: 0.84) and in each CIDP category except for DADS, in which peroneal nerves followed by tibial nerves were the most informative nerves.

Lower rate of demyelinating features as well as lower diagnostic accuracy (AUC: 0.78) of median with nerve respect to ulnar nerve could depend on the exclusion of prolonged DML from our analysis as a possible sign of carpal tunnel syndrome, when coupled with a consistent sensory conduction study. This certainly reduces the diagnostic potential of the median nerve and it should be properly considered when performing the electrophysiological exam.

On the other hand, the fact that the lower limb nerves had the most diagnostic accuracy in DADS patients is certainly consistent with a length-dependent neuropathy such as DADS neuropathy.

When facing a suspected CIDP patient, it is well known that if electrophysiological criteria for demyelination are not satisfied, the diagnosis as well as the access to treatment may be delayed or denied. Thus, in order not to miss early diagnosis of CIDP, several diagnostic criteria have been proposed and the most widely accepted criteria are those recommended by EFNS/PNS which appear to have the best combination of sensitivity (73%) and specificity (90%) for the diagnosis of CIDP [5–7]. Importantly, our 16% rate of patients not fulfilling the EFNS/PNS criteria is fully in line with sensitivity of applied criteria [6, 14]. Anyway, these patients have been considered affected by CIDP because of their clinical features together with supportive EFNS/PNS criteria. Indeed, we discussed clinical and laboratory features and we reconsidered a list of an alternative diagnosis, and collectively we reached a diagnostic consensus to minimize the possibility of a misdiagnosis [15–19].

Although expected, our analysis certainly demonstrated that the diagnosis of CIDP may be missed due to inadequate or incomplete electrophysiological examination or interpretation. Indeed, in possible and not-fulfilling CIDP patients, a lower number of motor nerves (median value 4 vs 6) and of TD (median value: 0 vs 4) measurements had been performed compared to definite and probable CIDP patients.

Consistently, the calculation of odds ratio revealed that each nerve added to examination increased the probability (OR: 1.59) to reach an electrophysiological diagnosis of definite and probable CIDP, especially when nerves of the upper limbs were tested (OR: 1.97). However, the investigation of more than 6 nerves did not improve further the diagnostic rate of CIDP.

In addition, for each proximal TD measurement, there was an additional 33% probability to reach an electrophysiological diagnosis of definite and probable CIDP, regardless of the number of tested motor nerves.

These data taken together could be useful to draw a thoughtful electrophysiological approach to patients suspected of CIDP. The joint task force of EFNS/PNS guidelines reports to test at first four nerves (median, ulnar, peroneal, and tibial nerves) on one side and, if criteria are not fulfilled, to study the same nerves at the other side.

Our findings suggest to start electrophysiological examination by testing ulnar and peroneal nerves bilaterally; afterward, taking into account the cut-off of six nerves, adding tibial nerves for DADS phenotype and median nerves for all other CIDP subtypes (typical, MADSAM, sensory and motor) could improve the electrophysiological diagnostic chance. Importantly, electrophysiological examination should consider all nerve conduction demyelinating findings including the evaluation of temporal dispersion.

However, distal CMAP amplitudes (<1mV) and especially from peroneal and tibial nerves may be too low for properly interpreting MNCV slowing as demyelinating [20, 21]. Moreover, both prolonged minimal F-wave latencies and absent F-waves may be classified as demyelinating features, but these findings are not specific, especially in the case of the peroneal nerve [20, 22].

Lastly, we are absolutely aware that distal CMAP duration can be very useful for CIDP diagnosis, but unfortunately, we could not consider it, as, given the retrospective nature of this study, filter settings to adjust the cut-off values were not available [23, 24].

## Supplementary information

ESM 1 (DOCX 19 kb)
