# Supplementary material for: The neurophysiological lesson from the Italian CIDP database
Source: Neurol Sci. 2021 May 21;43(1):573–82. doi: 10.1007/s10072-021-05321-z (PMC8724146; doi:10.1007/s10072-021-05321-z)
Supplement: Supplementary file 1 — (DOCX 19 kb) [file 10072_2021_5321_MOESM1_ESM.docx]

**Supplementary Table 1. Distribution of definite, probable, possible and not- fulfilling patients for each CIDP subtypes**

| **CIDP Subtypes** | **Definite and probable/Total (%)** | **Possible and Not fulfilling/Total (%)** | **Total, (%)** |
| --- | --- | --- | --- |
| **Typical** | 290/397, (73%) | 107/397, (27%) | 397/499 (79,7%) |
| **MADSAM** | 12/18, (66,7%) | 6/18, (33,3%) | 18/499, (3,6%) |
| **DADS** | 30/42, (71,4%) | 12/42, (28,6%) | 42/499, (8,4%) |
| **Pure Motor** | 10/20, (50%) | 10/20, (50%) | 20/499, (4%) |
| **Pure Sensory** | 7/17, (41,2%) | 10/17, (58,8%) | 17/499, (3,4%) |
| **Focal** | 1/5, (20%) | 4/5, (80%) | 5/499, (1%) |
| **Overall** | 419/499, (83,9%) | 80/499, (16,1%) |  |

**Supplementary table 2.** Electrophysiological features in patients not fulfilling the EFNS/PNS electrophysiological criteria

| Nerve | Motor nerve conduction velocity | | Distal motor latency | | F wave latency | |
| --- | --- | --- | --- | --- | --- | --- |
|  | N° tested | Mean ± SD | N° tested | Mean ± SD | N° tested | Mean ± SD |
| Right Median | 38 | 49.6 ± 7.1 | 39 | 4.1 ± 0.9 | 17 | 31.7 ± 3.6 |
| Left Median | 21 | 48.4 ± 6.4 | 22 | 4 ± 1.5 | 10 | 30.9 ± 2.3 |
| Right Ulnar | 50 | 50.8 ± 6.3 | 51 | 2.9 ± 0.5 | 31 | 32.3 ± 4.7 |
| Left Ulnar | 39 | 51.6 ± 7.6 | 35 | 2.9 ± 0.5 | 19 | 31.5 ± 4.0 |
| Right peroneal | 49 | 40.2 ± 6.4 | 50 | 5 ± 1.1 | 11 | 52.3 ± 10.3 |
| Left Peroneal | 47 | 41.5 ± 7.5 | 45 | 5 ± 1.3 | 9 | 56.2 ± 13.3 |
| Right Tibial | 32 | 39.1 ± 3.8 | 35 | 5.1 ± 1.1 | 14 | 59.8 ± 6.1 |
| Left Tibial | 34 | 38.9 ± 5.3 | 34 | 5 ± 1.3 | 16 | 58.7 ± 8.6 |

**Supplementary Table 3. Area Under the Curve (AUC) in ROC analysis for most informative couple of nerves in each subtype except for DADS**

| **Couples** | **Overall AUC** | **AUC in typical** | **AUC in MADSAM** | **AUC in pure sensor** | **AUC in pure motor** |
| --- | --- | --- | --- | --- | --- |
| **RU + LU** | 0,84 | 0,85 | 0,91 | 0,92 | 0,91 |
| **RM + LM** | 0,78 | 0,8 | 0,68 | 0,8 | 0,9 |
| **RP + LP** | 0,78 | 0,77 | 0,57 | 0,84 | 0,7 |
| **RT + LT** | 0,75 | 0,74 | 0,66 | 0,82 | 0,67 |
| **Most accurate couple of nerves** | | | | | |
| **RU + LP** | 0,86 | 0,84 | 0,82 | 0,95 | 0,81 |
| **RU + RP** | 0,85 | 0,86 | 0,80 | 0,89 | 0,82 |
| **RU + RT** | 0,85 | 0,85 | 0,81 | 0,92 | 0,75 |
| **RU + LT** | 0,84 | 0,85 | 0,80 | 0,89 | 0,77 |
| **LU + RP** | 0,84 | 0,84 | 0,71 | 0,96 | 0,90 |
| **LU + LP** | 0,83 | 0,83 | 0,75 | 0,8 | 0,85 |
| **RM + LP** | 0,83 | 0,83 | 0,70 | 0,8 | 0,8 |
| **LU + LT** | 0,83 | 0,83 | 0,76 | 0,78 | 0,82 |

RU: right ulnar; LU: left ulnar, RM: right median; LM:left median; RP: right peroneal; LP: left peroneal; RT: right tibial; LT: left tibial

**Supplementary Table 4. Area Under the Curve (AUC) in ROC analysis for most informative couple of nerves in DADS**

| **Couples** | **AUC in DADS** |
| --- | --- |
| **RU + LU** | 0,73 |
| **RM + LM** | 0,68 |
| **RP + LP** | 0,91 |
| **RT + LT** | 0,86 |
| **LP + RT** | 0,81 |
| **LP + LT** | 0,88 |
| **RP + RT** | 0,95 |
| **RP + LT** | 0,88 |
| **LP + LU** | 0,75 |
| **RT + LU** | 0,84 |
| **LP + RU** | 0,8 |
| **RP + LU** | 0,8 |

RU: right ulnar; LU: left ulnar, RM: right median; LM:left median; RP: right peroneal; LP: left peroneal; RT: right tibial; LT: left tibial
